# More-Than-Human: A Cross-Sectional Study Exploring Children’s Perceptions of Health and Health-Promoting Neighbourhoods in Aotearoa New Zealand

**DOI:** 10.3390/ijerph192416968

**Published:** 2022-12-17

**Authors:** Tiffany Williams, Kim Ward, Victoria Egli, Sandra Mandic, Tessa Pocock, Terryann C. Clark, Melody Smith

**Affiliations:** 1 School of Nursing, University of Auckland, Auckland 1023, New Zealand; 2 School of Sport and Recreation, Auckland University of Technology, Auckland 0627, New Zealand; 3 AGILE Research Ltd., Wellington 6011, New Zealand

**Keywords:** child-friendly cities, child wellbeing, participatory approaches, posthuman, public health, urban design

## Abstract

A disconnect between children’s ideas and their incorporation into environmental design, in the context of rapid urbanisation and climate crises, compelled us to reflect on children’s meaningful participation in positive environmental change. Our research aimed to bring new knowledge to the fore using a participatory, child-centred approach to understanding children’s perceptions of health and health-promoting neighbourhoods in Aotearoa New Zealand. The cross-sectional Neighbourhoods and Health study was conducted with 93 primary school-aged children (approximate ages 8 to 10 years) from two schools in Ōtepoti Dunedin and two schools in Tāmaki Makaurau Auckland from June 2020 to August 2021. We present a framework of twelve child-centred topics of importance for health (*Healthcare and ‘not getting sick’*, *‘How you feel’*, and *Taking care of yourself*), health-promoting neighbourhoods (*Proximity, safety and feel*, *Range of ‘places to go’*, *‘Friendly streets’*, and *‘No smoking’*), and those common to both (*Connections with other humans*, *Healthy food and drink*, *Exercising and playing sport ‘to keep fit’*, *‘Nature’ and ‘helping the environment’*, and *Recreational activities*). The more-than-human theory was used to situate our study findings, and we explored three threads evident in children’s thinking: (1) care for humans and non-humans, (2) vital interdependence of human–non-human relations, and (3) understanding complex urban environments through everyday activities. We conclude that the thriving of humans and non-humans in urban environments is important to children in Aotearoa New Zealand. We affirm that children have clear and salient ideas about health and health-promoting neighbourhoods.

## 1. Introduction

There is great potential—and great responsibility—when engaging in health and environment research with children. Despite children in middle childhood (ages 6 to 11 years) contributing important insights to topics such as health, their input is not necessarily taken seriously by decision makers [1]. The impacts of rapid urbanisation and climate crises will be inherited by today’s young people, compelling researchers and practitioners alike to reflect on how children’s meaningful participation can influence positive environmental change.

Firstly, we introduce the Neighbourhoods and Health study, describing children’s rights and child-centred approaches. We then highlight previous research from Aotearoa New Zealand, that has explored children’s perspectives on health and local environments. To situate the study findings, we draw on the more-than-human theory [2], described at the end of this introduction.

### 1.1. The Neighbourhoods and Health Study

The Neighbourhoods and Health study aimed to gather children’s current perceptions on health and wellbeing in urban neighbourhood environments in Aotearoa New Zealand (hereafter NZ), using participatory and child-centred approaches. The published study protocol includes a detailed session guide [3]. This research is situated within a children’s rights approach underpinned by the United Nations Convention on the Rights of the Child (UNCRC), of which Article 12 asserts that children have the right to express views and be given due weight in all matters affecting the child [4,5,6]. The Child Friendly City Framework for Action, developed to target the implementation of the UNCRC, advocates for cities where the voices, needs, priorities and rights of children are central to public policies, programmes, and decisions [7]. Reliance on the UNCRC as sole justification for children’s participation can dismiss other important guiding principles, such as national laws, human rights treaties, and research ethics guidelines, and can constrain deeper consideration of child participation [6,8]. The concept of child participation is contested on several levels through the domination of Global North ideals, socialising responsible citizens, and limited recognition of participation in children’s everyday lives [9]. A true child-centred approach to research actively questions adult assumptions about childhood [10]. In health and environment research, how adult researchers conceptualise children and childhood influences its design and outcomes [11,12]. The Neighbourhoods and Health study draws on the ‘new’ social studies of childhood [13]. This emergent paradigm positions childhood as a social construction that contextualises the early years of life, separate from biological immaturity. Children are positioned as competent social actors, in contrast to traditional views of children as passive subjects [14]. Key to our child-centred approach, participatory research methods are used to explore and prioritise children’s insights. Spyrou [15] asserts that researchers are responsible for exploring the limits of children’s voices by reflecting on the processes that produce them. Adult attitudes can limit children’s participation experiences based on adult understandings of age, competence, and voice [9]. Thus, a reflexive approach to child voice is needed in health-related research [16]. We acknowledge children’s participation in research as valuable and contested, position children as competent social actors, and offer a reflexive report recognising the need for ongoing critical examination of child participation.

### 1.2. Theoretical Framing: More-Than-Human Theory and Healthy Urban Environments

More-than-human, or posthuman, is an interdisciplinary theoretical perspective and relational ontology that moves beyond consideration of only humans and human agency to include non-humans [17,18]. Non-humans are entities and organisms that are present in everyday life and that shape society, such as animals, plants, weather, infrastructures, and technologies [2,19]. This theoretical perspective is especially pertinent to the field of geography, which resists intellectual division between the social and natural sciences [20]. Maller [2] reimagined healthy cities through a more-than-human lens as ‘…places and habitats that acknowledge, invite and encourage some non-humans to flourish and where beneficial more-than-human relations productively co-exist.’ (p. 152).

In their recent work with preschool-aged children in Ōtepoti Dunedin (hereafter Dunedin), NZ, on wellbeing affordances in the local environment, Ergler et al. [21] noted children’s engagement with the more-than-human world and ‘…the threaded togetherness of the wellbeing of all human and non-humans’ (p. 129). Maller [2] proposed that more-than-human thinking about healthy urban environments is challenging and risky but is a needed disruption to the status quo in the face of increasing urbanisation and a rapidly changing planet. Our use of this theory builds on perspectives from pre-school aged children in NZ [21] and allows us to rethink complex urban environments as more-than-human habitats, in a unique way not captured by other theories or models [2].

### 1.3. Children’s Perspectives on Health and Local Environments in Aotearoa New Zealand

Studies exploring NZ children’s perspectives about health have used methods such as focus groups, surveys, and participatory photography [22,23,24]. Children’s ages in these studies ranged between 6 and 13 years. Important concepts identified by children across studies included healthy food and drink (e.g., fruits, vegetables, and water), sport, recreation and exercise (e.g., scootering), being happy and around others (e.g., friends, family, and pets), having energy and sleeping well, and staying safe (e.g., sun safety, not feeling scared). Indeed, King and Cormack [24] concluded that health and wellbeing are conceptualised by children in holistic ways, and they acknowledged mokopuna (defined as ‘grandchild, grandchildren, descendant’ p. 377) Māori as knowledgeable experts capable of articulating views on their health, wellbeing, and environments.

Studies that have explored children’s perspectives on local environments in NZ are predominantly urban-based and have been undertaken with those aged 8 to 13 years. The Kids in the City (KITC) study [25] elicited experiences and the use of local neighbourhoods, including likes/dislikes, affordances for child independent mobility, and play [26,27,28]. The Neighbourhoods for Active Kids (NfAK) study explored children’s experiences of neighbourhood built environments [29]. The NfAK study identified that important destinations for children, such as parks, natural spaces, and shops, allow socialisation, active and imaginative play, along with consuming ‘unhealthy’ food and drink, and it highlighted concerns related to the safety of active school travel [30,31]. Ergler et al. [32] found that environmental opportunities and practical knowledge determined seasonal outdoor play, and independent play was possible in the suburban study location but not the inner-city location. Freeman et al. [33] reported that children clearly explain the natural world, connect with nature within their spatial and societal boundaries, and like being in nature.

### 1.4. Summarising the Knowledge Gap

Existing NZ research [22,23,24,25,26,27,28,29,30,31,32,33] affirms that children are capable of articulating important views on their health and local environments, such as common destinations, likes, dislikes, and affordances. Such research is often underpinned by a concern for child health and wellbeing [34]. Current understandings of health and urban environments using child-centred approaches are limited. We also note a paucity of research explicitly asking children about the interrelationship between their local environment and their health. Our research draws on children’s demonstrated capacity to articulate important views, and the need to advance current understandings of health and its relationship to the local environment (i.e., the neighbourhood) with children in urban NZ.

### 1.5. Aim

In this research, we aimed to understand children’s perceptions of health and health-promoting neighbourhoods in NZ using participatory and child-centred methods. In addition to topics of importance, we were interested in methods used and topics chosen by children for sharing with neighbourhood decision makers. We situate this research by drawing from the more-than-human theory, and we finally reflect on the implications of results for researchers and decision makers whilst considering children’s meaningful participation.

## 2. Protocol and Methods

The cross-sectional Neighbourhoods and Health study was conducted with primary school-aged children (approximate ages 8 to 10 years) in NZ between June 2020 and August 2021. The published study protocol includes a detailed session guide that covers set-up, materials, activities, and timing for two data collection sessions at each school, along with the research team’s credentials [3]. Two to four researchers of a team of six were present at each data collection session. The University of Auckland Human Participants Ethics Committee provided ethical approval (reference number 022910, 2019).

### 2.1. Setting

Our study was conducted in two cities in NZ. Stats NZ [35] identifies Tāmaki Makaurau Auckland (hereafter Auckland) as the country’s largest city, with a population of 1,571,718. The median age is 34.7 years, and the city is ethnically diverse (53.5% European, 11.5% Māori, 15.5% Pasifika, and 28.2% Asian). Auckland is located in northern NZ and is surrounded by an extensive coastline that houses multiple ports. Dunedin has a population of 126,255, with a median age of 36.8 years, and is less ethnically diverse (86.6% European, 9.3% Māori, 3.2% Pasifika and 7.8% Asian). Framed by hills and a natural deep-water port, Dunedin is a university town in southern NZ.

### 2.2. Recruitment

We used convenience sampling to invite four mixed-gender primary schools, two in each city, to participate. The convenience sampling method was selected to utilise existing relationships between schools and research team members in each city. To respect schools’ needs and preferences [36], we asked each school to choose a group of up to 30 students with no restrictions on age or school year level. A maximum of 30 students was stipulated to ensure an adequate researcher-to-participant ratio for our participatory and child-centred data collection methods. Three schools invited an entire class group, and one school invited a group of students from different classes who were members of a pre-existing school leadership group. The schools distributed student participant information sheets, parent consent forms and child assent forms, and collected these prior to data collection.

### 2.3. Data Collection Session One: Concept Mapping

Data collection session one focused on understanding children’s perceptions of health and health-promoting neighbourhoods. While seated in groups of four to six at tables or on the floor, children were invited to discuss, draw, or write ideas to answer ‘What does “health” mean to you?’. Responses on sticky notes were attached by participants to large sheets of paper (generation stage). Participant groups then sorted their ideas by grouping sticky notes in a way that made sense for them (sorting stage). Participant groups were asked to generate a statement for each grouping of sticky notes representing a key message (interpretation stage). Researchers assisted as required in writing statements that participants had generated verbally. Where drawings were unclear to the researchers, we asked children and wrote a description on the back of the sticky note. We repeated the process with the second question: ‘How does your neighbourhood help you and others to be healthy?’. Children were not given a predetermined adult-centric definition of ‘neighbourhood’, but rather they were encouraged to express their conceptualisations of neighbourhoods. A planned voting activity to determine priority statements for the whole group was not applied consistently across schools due to time constraints, so we did not analyse these data. In preparation for session two, we transcribed all ‘neighbourhood statements’ onto large sheets of paper. Responses to the final question, ‘How would you like to share your information with people that make decisions about your neighbourhood?’, were recorded by researchers during a whole group discussion to ensure that we had adequate and appropriate materials for session two.

### 2.4. Data Collection Session Two: Co-Creation of Dissemination Materials/Outputs

Data collection session two focused on children identifying and using creative methods to share their ideas, and on the topics of importance chosen when they were prompted to consider decision makers in their local neighbourhood. Participant groups reviewed all neighbourhood statements generated in session one and selected statement(s) to share using a creative method. Groups were free to use all materials provided (e.g., pipe cleaners and tablets with video software) and other classroom items. Three students across two schools chose to work individually. Researchers asked participants about what they were creating and took photographs of the outputs. At the end of session two, groups and individuals presented their final creations to the whole group. In each school, data collection sessions one and two were approximately two hours in duration. In one school, data collection was condensed into one session due to an impending COVID-19 lockdown. Following data collection, feedback materials were generated and disseminated to each participating school (see Figure S1, Supplementary Material).

### 2.5. Formal Data Analysis

Data related to ‘health’ and ‘neighbourhoods’, including ideas and statements from the concept mapping activities and drawing interpretations (e.g., a drawing of a hamburger recorded as ‘hamburger’), were captured using Microsoft Excel and QSR NVivo. Given the nature of the data (short comments with little contextual depth), we used a topic summary approach [37] and drew on reflexive thematic analysis concepts, as described by Braun and Clarke [38].

**Figure 1:**
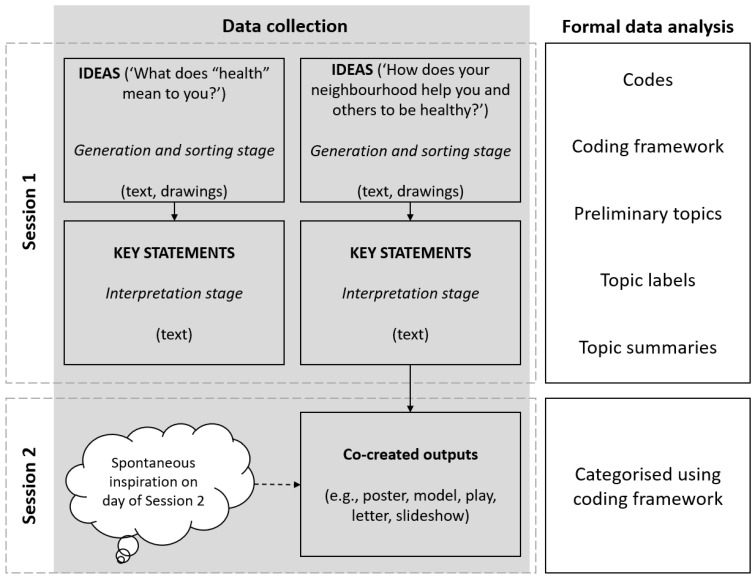
Visual summary of data collection and formal data analysis in the Neighbourhoods and Health study.

Topic summaries are conceptually different from themes and act as ‘buckets’ containing all the data related to a topic area [38]. Findings that are presented as topics, rather than themes, are appropriate where topics convey items of significance, add to what is already known, and do not attempt to describe an overall experience [37]. Identifying topics of importance to children for health and health-promoting neighbourhoods aligned closely with the study aim. Ideas were coded inductively, consistent with our intention of centring children’s perceptions, and we took a semantic-dominant approach that sought to represent meaning addressed explicitly by children [38]. Familiarisation by TW involved immersion in the data set through multiple readings, critical distancing, and questioning, and involved discussions with MS and KW as well as drawing on questions posed by Braun and Clarke [38] (p. 44) with a child-centred lens. Note making was a systematic process and included brief notes related to the whole data set. TW first coded all discernible ideas for health and health-promoting neighbourhoods and then coded key statements for health and neighbourhoods using preliminary topics. Following initial coding, TW discussed and adapted the coding protocol with MS and KW. In vivo codes are indicated by quotation marks. Children’s co-created outputs were categorised according to the method used and key topic(s) addressed using the coding framework generated through an analysis of ideas and key statements. A higher frequency of topics represented in co-created outputs did not necessarily indicate greater importance, but rather it allowed us to identify which topics participants wanted to share with local decision makers. Our analysis was not framed by the more-than-human theory. These perspectives arose through the process, and we used it to situate the study findings in our discussion. Spelling and minor grammatical errors were corrected for readability. Figure 1 provides a visual summary of the data collection and analysis.

## 3. Results

### 3.1. Participants

All four invited schools agreed to participate, and 93 children from these schools participated. Participants’ ages ranged between 8 and 10 years, and the number of participants in each school ranged from 19 to 28. One child did not participate due to the parent/caregiver not consenting and instead joined another class.

### 3.2. Ideas and Key Statements Generated by Participants

**Figure 2:**
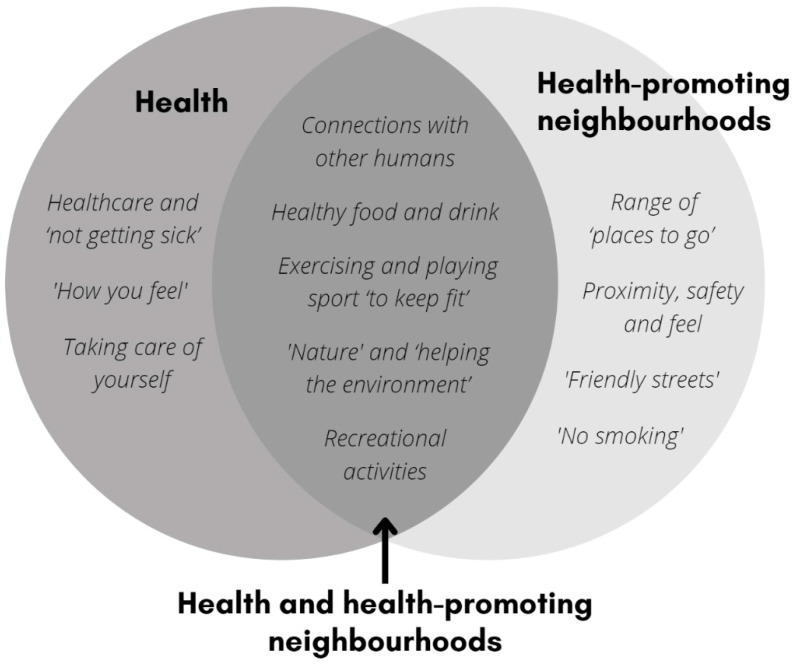
Framework of child-centred topics for health and health-promoting neighbourhoods.

Children generated a total of 613 (health) and 526 (health-promoting neighbourhoods) data points. Of the total data points, 2.5% (n = 29) were indiscernible and were coded as ‘meaning unclear’. Ideas about health and health-promoting neighbourhoods were provided predominantly in a text format (78% text and 22% drawings, and 77% text and 23% drawings, respectively). Children generated a total of 83 (health) and 75 (health-promoting neighbourhood) key statements. Figure 2 provides a framework of topics raised by children in relation to health, health-promoting neighbourhoods, and those common to both. Below, we summarise each topic.

### 3.3. Children’s Perceptions of Health

#### 3.3.1. Connections with Other Humans

For children, family (e.g., ‘family’ and ‘siblings’) and friends were the two groups of people central to health. Seeing, talking, and eating together with family were important, whereas children talked about seeing, playing with, and taking care of friends. Affection (‘hugs and kisses’) arose, although it was not related to any particular group of people. Helping others was featured strongly in a general sense (e.g., ‘to me health means helping others’), and in more specific ways. Specific ways of helping were primarily related to medical assistance, such as for ‘someone who got beat up’ and ‘bleeding people’. Participants’ perceptions of health were linked to ‘being kind’ and ‘doing kind things’. A connection between health and not dying, and possible collective responsibility for this, was hinted at through the idea of ‘saving lives’. Participants also acknowledged the need to help others in danger and to invite people to play if they were sad. In addition to helping other people, the importance of receiving help from others was also conveyed (e.g., ‘health means asking for help’).

#### 3.3.2. Healthy Food and Drink

Variations of ‘eating healthy’, ‘good food’, and ‘healthy foods’ were mentioned often, with frequency of eating healthily noted in some instances (e.g., ‘health means eating healthy food everyday…’). Eating healthily was connected with specific outcomes for the body (e.g., ‘vegetables make you brainy’ and ‘healthy foods keep your bones healthy’) and with feeling good (‘eating healthy keeps you energised and happy’). There was one mention of eating with other people (‘healthy family talk and eat together’). Fruit and vegetables were highly topical for children, both in general and in suggesting specific items. The phrase ‘5+ a day’ indicated knowledge of an educational campaign by a charitable trust in NZ that advocates for five or more servings of vegetables per day. Eating no or less ‘junk food’ was also important to children. Other generic terms were used (e.g., ‘fried food’, ‘takeaways’ and ‘fast food’). In addition to naming mainstream global fast-food chains, other specific foods included ‘pizza’, ‘lollies’, and ‘ice cream’. There was variation in the frequency that children thought such foods should be consumed, such as ‘do not eat…’, ‘eat less…’, and ‘only have […] sometimes’. This variation is illustrated by the subtle difference in these statements: ‘…eating treats and takeaways sometimes’ versus ‘…not eating sweets’. Hydration was important and was primarily connected to water, with or without reference to limiting fizzy drinks (e.g., ‘NO to coke, YES to water’). Limiting sugar was mentioned explicitly in two schools only (e.g., ‘don’t eat too much sugar’). Eating balanced meals and a variety of foods was connected with health, along with avoiding foods one is allergic to. There was little emphasis on the taste or enjoyment of food, with just one mention (‘eat yummy food’).

#### 3.3.3. Exercising and Playing Sport ‘to Keep Fit’

Exercise was central to children’s perceptions of health. Getting and keeping fit were also topical, illustrated by ‘health means to stay fit and exercising’. Few explicit examples of how children understood exercise were offered. A notable exception was the statement ‘exercising to us means climbing trees, gym, biking, and swimming’, which indicated a broad conceptualisation of exercise in one instance. Other general terms used for being physically active were ‘working out’, ‘being active’, and ‘keep moving’. A wide range of physical activities was mentioned. Running, jogging, and walking were the most common, with walking often linked to walking dogs. Relatedly, children’s ideas of who should undertake regular exercise extended beyond themselves to include pets (‘health means exercising you and your pets often’). Variations of ‘weight training’ and ‘weight lifting’ were mentioned several times, as was ‘going to the gym’. A wide range of pursuits that we categorised broadly as sports was highlighted (e.g., ‘cricket’, ‘gymnastics’, and ‘netball’). Such pursuits may reflect recreation rather than sport, but without that context from the children, they were located within exercise and sport. There was only a brief mention of outcomes related to exercise and sport, namely linking exercise with body size and sport with happiness.

#### 3.3.4. ‘Nature’ and ‘Helping the Environment’

Children reported natural features concerning health, which were trees and plants, air and water quality, and animals. Trees were important to children (e.g., ‘green trees’), as well as ‘not cutting down trees’ and ‘planting trees’. The terms ‘clean’ and ‘fresh’ were used interchangeably in relation to both air and water. Air quality was a particular focus, for example, ‘being healthy means getting fresh air’ and ‘health means breathe clean air…’. Air quality was linked to both breathing and the brain. ‘Animals’ were mentioned frequently by children, including ‘keeping animals safe’. There was less emphasis placed on being in nature (e.g., ‘health means to go and be in more nature’) than on the presence of numerous natural features described above. The importance of ‘helping the environment’ was evident, illustrated by ‘health means keeping the environment healthy’. How waste is managed was important to children, with ‘no plastic’, ‘no littering’, ‘recycling’, and ‘start upcycling’ in particular. The interrelationship of individual human health and the environment was made explicit by the following: ‘no littering because it’s bad for your mental health and the environment’. This perception was otherwise implicit; when children were asked what health meant to them, they referred to numerous ways to take positive environmental action. Additional examples of positive environmental action were ‘use electric cars’ and ‘growing organic food’.

#### 3.3.5. Recreational Activities

Recreational activities were central to children’s perceptions of health, in particular going outdoors (e.g., ‘go outside in the sun’). Going outdoors was implied by multiple references to getting fresh air, for example, ‘get fresh air for [your] brain’. Without additional context, it is impossible to know what kinds of outdoor settings children were referring to, but we can observe that spending time outside is valuable to children. ‘Playing’, in general and with friends, was also important. A wide range of ways to spend leisure time was described. Popular activities were reading books, climbing trees, jumping, and gardening. Other activities mentioned multiple times, but only in one school each, were riding bikes and scooters, music, dance and time alone. Music and dance were the only activities that children connected to particular outcomes: ‘music is healthy because when you listen to it, it’s relaxing’ and ‘dancing is healthy because it keeps you fit and it’s fun’. Only one other reference was made to fun (‘having fun’). Moderating use of technology (i.e., computer games, video games, and television) was featured strongly in relation to health. Perceptions ranged from no acceptable level of use (e.g., ‘don’t play video games’, ‘no computer games’) to limiting use (e.g., ‘health means not playing too much video games’). The statement ‘balance technology and going outside’ illuminated children’s views on mediating time between stereotypically indoor activities and outdoor pursuits.

#### 3.3.6. Healthcare and ‘Not Getting Sick’

Children’s emphasis on health as being related to sickness reflected a clear understanding of biomedical perspectives on health. Healthcare was mentioned consistently, in particular in medical and dental care with doctors and dentists. Treatment of current health issues was a focus for children (e.g., ‘go to the doctor when sick’ and ‘going to dentist if you have bad teeth’). Seemingly contradictory views of ‘go to the doctor’ and ‘not going to the doctor’ were pervasive in children’s perceptions of health. The following illustrate this idea: ‘health means an apple a day keeps the doctor away’ and ‘being healthy means going to the doctors’. These seeming contradictions may reflect that health means ‘not getting sick’ and therefore not needing to go to the doctor, or that going to the doctor should be done routinely for ‘staying healthy’. Regardless, doctors were highly relevant to children’s perceptions of health, and hospitals were referenced frequently. The absence of specific conditions (e.g., ‘hypothermia’ and ‘flu’) and symptoms (e.g., ‘throwing up’) were linked to health, but children also highlighted the inevitability of sickness (e.g., ‘bad and good health’ and ‘becoming sick’). ‘Not dying’ was also linked with children’s perceptions of health. ‘Take vitamins’, ‘tablets’, and ‘medicine’ were important, as illustrated by ‘health is having lots of vitamins and medicines to stay healthy’. There was a strong emphasis on preventing the spread of sickness, with the most common measure of staying at home if you are sick, alongside other measures (e.g., ‘cover mouth when sneezing/coughing’, ‘COVID mask’, and ‘hand sanitising’). Physical parts of the body (e.g., ‘heart’ and ‘bones’) and physiological processes (e.g., ‘burn calories’ and ‘hearing’) were of interest, as was their optimal state (e.g., ‘healthy heart’). The COVID-19 virus was mentioned explicitly on several occasions, such as ‘make sure COVID goes away’ and ‘health means killing the coronavirus’.

#### 3.3.7. ‘How You Feel’

Children’s perceptions linked health with ‘how you feel’ and ‘mental health’. Limited insight was provided into how children understood mental health, but one key statement highlighted the role it could potentially play in displaying emotion: ‘mental health can keep you healthy because you are not crying’. Feeling ‘happy’, ‘positive’, and ‘calm’ were mentioned consistently. Being happy was directly related to health (e.g., ‘keeping myself healthy and happy’ and ‘healthy feelings—happy’), as was the happiness of friends and family. Positivity was referenced directly (e.g., ‘thinking positive’ and ‘feeling positive’) and indirectly (e.g., ‘feeling good about yourself’ and ‘don’t give up’). Staying calm was also important, as, put simply, ‘health means to stay calm’. Feeling good in general was desirable, illustrated by ‘health means feeling good’. The relevance of ‘strength’ and ‘staying strong’ was consistently linked to health (e.g., ‘health means being happy and strong’). It was unclear what being strong meant to children without additional context, but this idea was related to the body (‘keeping body strong’) and feelings (‘feeling good and strong’).

#### 3.3.8. Taking Care of Yourself

Taking care of yourself and of your body was topical for children, as said in ways such as ‘look after your body’ and ‘protecting your bodies’. In one instance, this extended to others (‘take care of friends/yourself’), but it usually referred to things that they would do for themselves, namely personal hygiene, sleep, and relaxation. Emphasis on ‘good hygiene’ was strong and included washing hands, brushing teeth, nose blowing, and bathing. The importance of personal hygiene to children’s perceptions of health was exemplified by statements such as ‘health means to have good hygiene to stay healthy’ and ‘being healthy means being clean’. Sleep was pertinent for children, referring to both quality (e.g., ‘getting good sleep’ and ‘sleeping normally’) and amount of sleep (e.g., ‘getting 10 h sleep for health’ and ‘get lots of sleep’). Rest was mentioned in a general sense, such as ‘resting when needed’, and was associated with sleep, for example, ‘sleep to get rest’. ‘Relaxation’ was also topical (e.g., ‘taking relaxing time’). It was linked in one instance with reducing worrying (‘relax brain and not worry’), and, in another, with certain activities (‘relaxation to us is books and riding trains’).

### 3.4. Children’s Perceptions of Health-Promoting Neighbourhoods

#### 3.4.1. Connections with Other Humans

A health-promoting neighbourhood was one that ‘helps you be social with others’ and ensures ‘we are never alone’. The groups of people that children mentioned concerning this topic were family, friends, and neighbours. Family included siblings and cousins, and time together was spent playing and getting fresh air. Friends were viewed as companions for playing, biking, and walking. Of particular importance were friends who lived nearby or ‘down the road’ and the role of friends in ‘making you happy’ and ‘taking care of you when you’re feeling down’. In regard to neighbours, children’s perceptions ranged from knowing your neighbours and having polite interactions (e.g., ‘saying “hi” to neighbours’) and day-to-day engagement with neighbours (e.g., exercising, playing, and mowing lawns) to sharing special occasions (e.g., ‘on Christmas, I climb over to give my neighbour presents’). Important qualities of neighbours were ‘friendly’, ‘good’, ‘nice’, and ‘reliable’. In general, important qualities of people in a health-promoting neighbourhood were those who were ‘kind’, ‘helpful’, ‘happy’, and ‘trustworthy’. Neighbours who ‘always have a smile’ were referenced, hinting at the value of positive interactions for children. Other polite interactions in the wider neighbourhood, such as knowing, greeting and talking to others, were also mentioned by children. Helping others was featured strongly and was mostly understood in broad terms (e.g., ‘help each other when needed’ and ‘look after people and care for them’), but specific examples were also offered, such as repairing houses, in times of distress, when toys get lost, and during lockdown (assumed as being related to COVID-19). Elderly people were the only group that children specifically mentioned helping, such as ‘help older people’ and, more specifically, ‘go shopping for elderly people’. Being able to receive help when needed was also mentioned, as was ‘helping pets’, for example, ‘cat or dog sitting’. The giving and receiving of food were topical for children, for example, receiving food at someone’s home, giving and receiving baked food, being offered food, giving away fruit from your tree, donating food to help others, the availability of ‘community food’, and ‘free food storage’. The statement ‘if you got things that keep people healthy and you got too much of it (such as fruits), give it away to others’ illustrates children’s perception of sharing food as one way of helping others in a health-promoting neighbourhood. Children saw people in their neighbourhood as having a role in their personal safety, for example, ‘it is nice to have trusted and kind neighbours to keep you safe’ and ‘keep you safe when around nice people’.

#### 3.4.2. Healthy Food and Drink

Eating healthily was featured but with limited emphasis regarding health-promoting neighbourhoods. Good food and healthy food were mentioned in a general sense. The importance of ‘getting healthy food nearby’ was noted, such as ‘getting veggies from [the] dairy’ (in NZ, ‘dairy’ commonly refers to a small mixed grocery store). Fruit and vegetables were conceptualised in regard to neighbourhoods as ‘plant[ing] vegetables’ and ‘…having big healthy veggie gardens’, as well as fruit trees both at home and in the street. There was only one mention of junk food (‘discourage us from junk food’), which could be understood as children perceiving a health-promoting neighbourhood as one that discourages the consumption of junk food. One statement (‘farmers make food to stock up supermarkets’) illustrated consideration of food systems and the role of specific actors in food supply chains.

#### 3.4.3. Exercising and Playing Sport ‘to Keep Fit’

Specific sports (e.g., ‘badminton’, ‘cricket’ and ‘soccer’) were perceived by children as important, with occasional mention of sports clubs (e.g., ‘gymnastics club’). Sometimes the context for where the sport would be played, namely ‘gymnastics down the road’ and ‘play basketball next to my garage’, was indicated. Exercise was mentioned sporadically with context about where (e.g., ‘leisure centre to exercise’) and with whom (e.g., ‘exercise with neighbours’). The statement ‘exercising, walking, and biking together’ highlights other people as an important feature of being active. Running had lesser emphasis than walking in regard to health-promoting neighbourhoods. ‘Having places to walk’ was important, such as to school and around the neighbourhood, block, and street. Walking with dogs or with friends was also important. The statement ‘my neighbourhood is healthy because it is easy to walk to school and run around’ illustrated the ease of moving around on foot as important in health-promoting neighbourhoods.

#### 3.4.4. ‘Nature’ and ‘Helping the Environment’

Trees were central to children’s perceptions of health-promoting neighbourhoods, exemplified by ‘healthy neighbourhood means having lots of trees’. Suggestions of planting more, letting them grow, not cutting them down, and keeping them healthy reflected the centrality of trees. Plants, bushes, flowers, and grass were all important, as were woods and forests. The relevance of air quality was shared in different ways, from ‘not use many cars for clean air’ to ‘fresh oxygen’ and ‘less pollution’. Oxygen was a desirable element, for example, ‘if we have more nature, it will produce more oxygen’. In relation to how neighbourhoods help us and others to be healthy, animals and pets arose frequently. Lots of cats, dogs, and animals were desirable, as were ‘cool birds’. Walking dogs and playing with cats were highlighted, in addition to ‘seeing’, ‘helping’, and ‘loving’ pets. Children briefly mentioned other natural features, namely ‘mountains’, ‘hills’, ‘river’, and the ‘ocean’. Children offered a holistic view incorporating multiple elements of nature, for example, ‘Fresh and healthy water to drink. Good to have lots of plants and trees. Plants and trees for oxygen and grass for the animals…’. Taking positive environmental action was topical to health-promoting neighbourhoods. As well as planting and taking care of trees, children cited several ideas related to rubbish, such as ‘more rubbish bins’ and ‘no littering’. Less plastic, picking up plastic, and making beaches clean were also mentioned, as was using electric cars and less cars in general. ‘Less cars on roads, so we don’t pollute the air more. So, more bikes and less cars’ illustrated a link between cars and less pollution or clean air.

#### 3.4.5. Recreational Activities

Recreational activities were featured strongly for children. A neighbourhood that supports health was one that ‘gets you out of the house’. ‘Playing’, and to a lesser extent ‘fun’, were important, illustrated by the statement ‘having fun and playing outside makes you healthy’. Playing with siblings, friends, neighbours, and pets was mentioned, in addition to playing at the playground and outside. Having fun was linked to playgrounds, parks, and climbing trees. Biking, scootering, and skateboarding were popular. Biking was seen as healthy and something to do with friends; scootering was something you did to school. A neighbourhood with places and spaces that enabled biking and scootering was desirable, illustrated through statements such as ‘more space to bike’ and ‘good neighbourhoods have good places to scooter’. Trampolines and trees were related to jumping and climbing, respectively. Water-based activities included ‘pontoon swimming’, ‘fishing’, and ‘kayaking’. Activities that were mentioned several times, but only in one school each, were ‘camping’, ‘having a campfire’, and ‘gardening’.

#### 3.4.6. Proximity, Safety, and Feel

Children spoke clearly to three specific elements for health-promoting neighbourhoods: proximity, safety, and feel. A reference to the closeness of people and places conveyed the importance of proximity. Having friends who ‘live nearby’ and ‘down the road’ was desirable, as was being close to school and being able to walk or scooter to school. Other features children liked close by were sports (e.g., ‘gymnastics down the road’), play opportunities (e.g., ‘local playground’), healthy food (e.g., ‘plum tree at my house’), and natural features, such as ‘living near the ocean.’ Both ‘feeling safe’ and ‘safe place[s]’ were consistent safety considerations for children. Feeling safe was only spoken about in general terms (e.g., ‘you feel safe’), whereas safe places were described in more detail, such as ‘safe places in our environment to play—green park, playground’ and ‘healthy neighbourhoods have safe place to bike and scooter’. ‘Safe’ and ‘safer’ roads were important to children. ‘Trusted and kind neighbours’ and ‘nice people’ had a role in keeping children safe. The feel of a health-promoting neighbourhood was described using a wide range of adjectives, such as ‘kind’, ‘friendly’, ‘caring’, ‘happy’, ‘energetic’, and ‘colourful’. The statement ‘energetic things to do—kayaking, soccer field, gym, speedway…’ illustrated how children may experience the feel of a neighbourhood through various activities.

#### 3.4.7. Range of ‘Places to Go’

Children mentioned a diverse range of places. A health-promoting neighbourhood was one with ‘nice places’ to visit that were plentiful (e.g., ‘having a lot of places to go’). Places with the strongest emphasis from children were parks, playgrounds, shops, and beaches. Lots of parks, which were big and had ‘equipment’, were ideal, as were fun playgrounds for kids and ‘building more playgrounds’. The shops that were mentioned were primarily food stores (e.g., ‘milk shop’ and ‘food store’) and supermarkets. The beach was noted as ‘a great place to swim’. Other places that were important to children were gardens, places to bike/scooter/skate, schools, and homes. ‘Lots’ and ‘bigger’ gardens were wanted, specifically vegetable gardens (e.g., ‘get bigger veggie gardens’). Places to bike, scooter, and skate were topical, for example, ‘more bike tracks’ and ‘big scootering place’. Schools were mentioned without any extra detail, and housing was referenced only in general terms (e.g., ‘good homes’ and ‘nice houses’). Additional places mentioned were churches, gyms, and speedways. Insight from children into the role of different places was limited but hinted at the importance of play (e.g., ‘healthy neighbourhood gives us parks to play in’ and ‘…parks make us have a lot of fun…’) and social connection (e.g., ‘halls and churches to meet people’).

#### 3.4.8. ‘Friendly Streets’

Transportation, particularly transport infrastructure, was important to children in health-promoting neighbourhoods. The need for ‘more traffic lights’ and ‘better footpaths’ was highlighted, and in one school, ‘zebra crossings’ were mentioned. Roads were of particular interest, especially ‘less traffic on roads’ and ‘safer roads’. Speeding was also pertinent (e.g., ‘stop speeding’), with suggestions for speed signs (e.g., ‘speed sign (40 km)’) and speed humps. Perceptions regarding cars were related to using electric cars and fewer cars to reduce pollution. Children’s consistent use of active words such as ‘more’, ‘less’, and ‘stop’ was apparent, indicating a potential interest for change around the topic of ‘friendly streets’.

#### 3.4.9. ‘No Smoking’

References to smoking came from only one school but multiple times. Related to health and neighbourhoods, children from this school suggested stopping various groups from smoking (‘teenagers’, ‘parents’, and ‘grandparents’), in addition to ‘banning cigarettes and vaping’ and ‘telling people not to smoke’. These views were summarised as ‘healthy neighbourhood means there is no smoking’.

### 3.5. Co-Created Outputs for Health-Promoting Neighbourhoods

**Figure 3:**
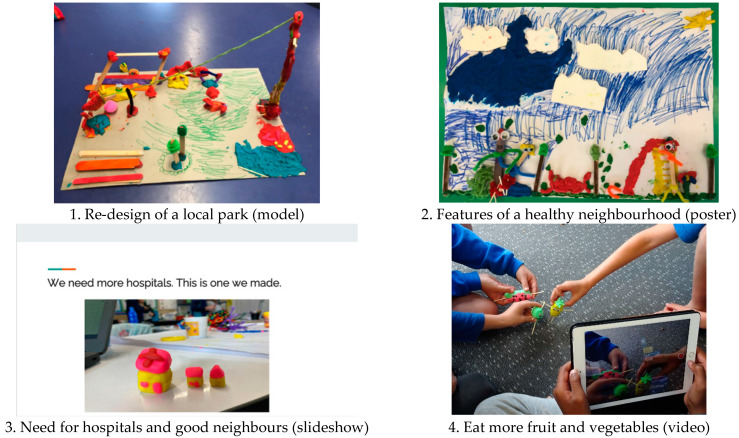
Examples of children’s co-created outputs in the Neighbourhoods and Health study.

A total of 24 co-created outputs were generated by participants across all schools in data collection session two. Of particular interest to researchers were the types of creative methods that children used and the topics they chose to share for an audience of decision makers in their neighbourhood. The range of methods selected by participants were as follows: video (n = 8), slideshow (n = 7), combined poster and model (n = 3), poster (n = 2), model (n = 1), letter (n = 1), map (n = 1), and a play (n = 1). Co-created outputs featured a range of different topics, and some outputs featured more than one topic. Coded using the child-centred topics of importance, the frequency of topics that were shared was as follows: *‘Nature’ and ‘helping the environment’* (n = 6), *Range of ‘places to go’* (n = 6), *‘No smoking’* (n = 4), *Exercising and playing sport ‘to keep fit’* (n = 3), *Connections with other humans* (n = 2), *Healthy food and drink* (n = 2), *Recreational activities* (n = 1), and general features of a health-promoting neighbourhood (n = 3). Figure 3 provides selected examples of children’s co-created outputs.

## 4. Discussion

This research aimed to understand children’s perceptions of health and health-promoting neighbourhoods in NZ using participatory and child-centred methods [3]. We present a child-centred framework of twelve topics that were important to children aged 8 to 10 years (Figure 2). Children produced a total of 24 co-created outputs. Their most favoured output methods were videos and slideshows; the most popular child-centred topics they shared were *‘Nature’ and ‘helping the environment’* and *Range of ‘places to go’*. Children were able to conceptualise ideas of importance in multiple and interrelated ways, solidifying the positioning of children as competent social actors and building on the known importance of child-centred approaches to garner unique insights [39]. Overall, children expressed clear and salient ideas about health and health-promoting environments.

### 4.1. Children’s More-Than-Human Thinking

We highlight more-than-human theoretical perspectives as a useful means to situate our child-centred topics of importance, and to bring our study findings to life in a novel way. The more-than-human theory came to the fore when we identified that children’s perceptions went beyond the human self to include myriad non-human others, such as trees and animals. Previous NZ research highlighted preschool-aged children’s engagement with more-than-human aspects of their surroundings [21] and the interconnectedness of humans and non-humans in imaginary cities they created [34]. We offer three related threads to illuminate the presence of children’s more-than-human thinking in the Neighbourhoods and Health study: (1) care for humans and non-humans, (2) vital interdependence of human–non-human relations, and (3) understanding complex urban environments through everyday activities.

Firstly, the importance for children of being in or near ‘nature’ was superseded by actively caring for and protecting non-human others, such as plants and animals. Similar to research with mokopuna Māori that found a link between their own wellbeing and the wellbeing of the environment [24]; children in our study explicitly linked the concept of health to keeping the environment healthy. Exercise was described as something that you needed for yourself and that is required by pets. In the context of healthy urban environments, Maller [2] proposed that drawing on the principles of care for humans and non-humans is central to more-than-human thinking. From their research with pre-school-aged children in NZ, Ergler, Freeman, and Guiney [34] suggested the use of ‘care-full’ urban environments, where children are both recipients of care and care agents at multiple scales. Our findings add weight to the view that children value the thriving of all species in urban environments.

Secondly, children demonstrated an awareness of human–non-human relations that was deeper than simple interconnectedness and more akin to vital interdependence. We draw on children’s words to bring this thread to life: ‘Birds spread the seeds. There is space to walk and run. Nature and plants give us air. Animals give us food. Flowers keep the bees alive so we get honey. Weather gives us rain to grow crops.’ In this example, children have seamlessly woven the various needs and contributions of human and non-human entities. We note human-centredness in this quote, but, as described further below, children are constrained by the language of dominant discourse. Children’s vivid portrayal of the multiple interdependences that exist between and within humans and non-humans in urban environments demonstrate more-than-human thinking [2].

Thirdly, multi-faceted everyday activities were highly pertinent to children regarding health and health-promoting environments. In advancing their argument for more-than-human perspectives for healthy urban environments, Maller [2] highlighted social practice theories as imperative for re-thinking cities as sites, comprising ‘…extended and interlinked material arrangements and the activities and doings carried out by humans and non-humans’ (p. 72). Many activities described by children reflected social practices of their everyday life, such as walking the dog, climbing trees, and scootering to school. Through focusing on multi-faceted everyday activities, children invite us to reconsider dominant models of health behaviour where individual agency and/or external factors result in ‘healthy’ or ‘unhealthy’ behaviours. Rather, health is shaped by the social practices of everyday life, and these practices offer valuable insight into more-than-human relations in urban environments [2].

The human-centredness, and nature/environment versus human binaries, that were discernible in children’s words may appear to contradict the proposed threads of more-than-human thinking. A limitation of child voice is that children draw from inherited language and speech; what children offer researchers through their words is mediated by discourses that are available to them [15]. Given that human-centredness and binary understandings dominate current discourse, it is not surprising that children at times replicated this view. In the current study, children were also able to draw their responses, as the use of multiple methods is an enabler of child voice [39]. To conclude, we reflect on the following question posed to children: ‘How does your neighbourhood help you and others to be healthy?’. The open framing, without an adult-centric definition of ‘others’ (or ‘neighbourhood’), evidently gave children the freedom to share about important humans and non-humans in their worlds. This further solidifies the importance of child-centred methods in health and environment research.

### 4.2. Implications of Neighbourhoods and Health Study Results

Our results and discussion of the Neighbourhoods and Health study begs the question, ‘what now?’. We consider two key groups, researchers and decision makers (e.g., practitioners and policy makers), and link our results to the broader conversation on children’s meaningful participation.

For researchers, these results can help develop questions and measures that capture understandings of health and health-promoting neighbourhoods from children’s perspectives. Future studies could use the child-centred topics identified in this study as a starting point, in preference to adult-centric measures, and could consider the creative methods that children selected to share their ideas. Reports from previous NZ research attest that children offer unique insights regarding cities. Examples include children (aged 3 to 4 years) identifying city features (e.g., people) that adult researchers had inadvertently overlooked [40], adult researchers’ perceptions of mundane elements in urban environments changing through engaging with children (aged 9 to 12 years) regarding play in public places [28], and child researchers (aged 10 to 12 years) asking questions that adult researchers had not considered (e.g., why children were expected to pay for using sports facilities when they had no money) [41].

For decision makers, especially practitioners, the methods described in this article and the published session guide [3] provide a practical toolkit for exploring ideas with children. Policy makers are offered current perceptions from a sample of children in middle childhood in two geographically and ethnically diverse cities of NZ. We plan to share the Neighbourhoods and Health study results directly with local decision makers, such as city councils.

Hunleth, Spray, Meehan, Lang, and Njelesani [1] advocated that the cornerstone of children’s meaningful participation is listening without tokenising or diminishing their perspectives and experiences. Their recent scoping study found that children’s meaningful participation in health research has not increased with time [1]. Previous NZ research flags implications for decision-makers, such as local councils and planners, regarding knowledge gained through urban environment research with children [26]. It also notes the challenges of effectively translating such knowledge to resource allocation and practice [27,42]. Additionally, Spray [10] suggested that well-meaning researchers and policy makers are unsure how to include, interpret, and apply children’s perspectives about health. These NZ perspectives join a global evidence base that suggests a disconnect between children’s ideas and their incorporation into environmental design, planning, and implementation [12,43,44]. Researchers may do well to embrace collaborative processes from practice, such as co-design, a design-led process, which can facilitate children’s participation in addressing real-world concerns [11]. Decision-makers may benefit from drawing on participatory child-centred methods and learnings from research, such as the reflective guide for child-centred thinking in health intervention research [1]. Foregrounding participatory processes with models of child participation can enhance the process and optimise outcomes, for example, the framework offered by Lundy [45] for conceptualising Article 12 of the UNCRC [11]. The clarity and saliency of children’s ideas in the current study, coupled with a lack of improvement in meaningful participation and disconnect between input versus outcomes, highlight the translation of children’s ideas for health-promoting environments as requiring further investigation.

### 4.3. Reflexive Account of the Research Process

Reflexive research with children ‘…accepts the messiness, ambiguity, polyvocality, non-factuality and multi-layered nature…’ (p. 162) of meaning produced, and pays attention to context and power [15]. Our flexibility in the Neighbourhoods and Health study process reflects an ethically informed approach [36]. We were required to modify the timing and flow of activities, even with a study-specific session guide. This messiness was felt by researchers as a balancing act between meeting the emergent needs of participants and the school setting, whilst staying true to planned activities for consistency between schools. Children’s insights were welcomed in a range of forms. As researchers, we found it challenging to document, integrate, and (re)represent data sources that were linked yet unique, but it was inspiring to observe children’s level of insight and their joy at the creative freedoms offered. Data collection and analysis required that we embraced ambiguity. Ideas not discernible to researchers during data analysis were coded as ‘meaning unclear’, which raised the question of what it means when we have data we cannot code. It is possible that our methods limited adequate context for responses, that children were disengaged from the questions posed, or other reasons. Interpreting what children say is logistically and intellectually challenging, and children actively construct their social worlds [10]. Our analysis and writing process strived to represent the breadth and depth of participants’ responses and to summarise what we heard. The outcome was the unification of diverse participant voices gathered at different time points across four locations. Visual methods, such as the ones used in our study, move beyond reliance on interaction with an interviewer. Such methods allow children to express themselves in a myriad of ways, but, as highlighted by Spyrou [15], outputs from visual methods (e.g., images) are only selections from numerous possibilities. Thus, any interpretations of images by researchers or children are both positioned and selective [46].

We draw attention to two pertinent elements regarding study context: undertaking the Neighbourhoods and Health study in a school setting and during the COVID-19 global pandemic. Schools are adult-controlled settings known to enable or inhibit children’s participation [16]; we experienced both concurrently. Schools enabled assent/consent processes, they afforded us protected time with a consistent group of children, teachers assisted during sessions, classroom resources such as laptops were available, and children appeared relaxed in their familiar setting. However, children who were invited to participate were selected by the schools, inhibiting our ability to provide an equal opportunity to all children. The adult–child hierarchy was assumed and difficult to disrupt. Participants were isolated from intergenerational social relationships, such as parents and siblings, and navigating non-participation was difficult due to classroom expectations that children are involved in all activities. Teachers upheld behavioural expectations such as sharing, which facilitated progress, but this may have also limited more expressive ideas or outputs. Research with children calls for close consideration of power relations [15]. Researchers play an important role in ensuring meaningful participation at all stages of the process, and there is a delicate balance between centring children and bringing expert guidance. We felt this most acutely when prompting discussion, as we strived to ensure children’s understanding of the activity whilst not influencing their responses.

Our study happened amid the COVID-19 global pandemic, which may have influenced what was important to children. We noted explicit (e.g., ‘COVID mask’) and likely implicit (e.g., ‘cough into your arms’) references to COVID-19. Participants may have emphasised topics pertinent to children in NZ during COVID-19, such as neighbourhood social connections, natural environments, slowing down, time with family, kindness, and caring for others [47,48].

### 4.4. Strengths and Limitations

Participatory and child-centred approaches were notable strengths of the study. Drawing from our recent review on participatory processes with children, we described our child-centred research process, how children and childhood were conceptualised, the role of all groups involved, and the ages of child participants [11]. Children communicated ideas through writing, drawing, and verbally clarifying and were able to co-create an output using a method of their choosing. We engaged with children from two geographically and ethnically diverse cities and with those in middle childhood who were known to have fewer opportunities to share their perspectives on health [1] and urban planning [42].

The nature of the data, short comments with little contextual depth, may have limited meaning. Our situated and contextual findings reflect what was relevant and important to participants. A limitation is that all topics of interest to researchers and/or decision makers may not have been discussed by participants. For example, specific qualities of neighbourhood facilities were rarely noted by children. In contrast, when considering environments such as streets, numerous comments were made about specific streetscape qualities. Longer or more involved participatory research with children could present more complex understandings, for example, nuanced understandings related to specific qualities of neighbourhood facilities, but these would not necessarily be more true or authentic [15]. Moreover, if we worked with more or different groups of children, alternative insights could have arisen. Nonetheless, this research makes important contributions to planning health-promoting neighbourhoods through providing a framework of child-centred topics of importance, novel insights around children’s more-than-human thinking, and detailed descriptions of the participatory methods that we used. Our findings are not representative of children across NZ, nor were they intended to be; rather, we aimed to provide updated perceptions from children in urban areas about health and health-promoting neighbourhoods using a fit-for-purpose session guide.

## 5. Conclusions

This research sought to bring new knowledge to the fore using a participatory child-centred approach. The presented framework summarises child-centred topics for health and health-promoting neighbourhoods and has relevance for researchers and decision makers. Our findings corroborate the need for a shift around dominant adult understandings of health, wellbeing, and health-promoting urban environments towards more-than-human perspectives. This involves exploring how we de-centre humans and how we design, plan, and implement from a place that honours human and non-human entities. Our findings indicate that, for middle childhood-aged children in urban NZ, the thriving of humans *and* non-humans underpins human health and wellbeing. Participants in this study affirmed that children in NZ have clear and salient ideas about health and health-promoting neighbourhoods. We advocate that children require meaningful participation opportunities in research and practice that, beyond sharing ideas, are valued in decision-making processes.

## Supplementary Materials

The following supporting information can be downloaded at: https://www.mdpi.com/article/10.3390/ijerph192416968/s1.
