# Supplementary material for: More-Than-Human: A Cross-Sectional Study Exploring Children’s Perceptions of Health and Health-Promoting Neighbourhoods in Aotearoa New Zealand
Source: Int J Environ Res Public Health. 2022 Dec 17;19(24):16968. doi: 10.3390/ijerph192416968 (PMC9779710; doi:10.3390/ijerph192416968)
Supplement: Supplementary file 1 [file ijerph-19-16968-s001.zip › ijerph-2069328-supplementary.pdf]

SUPPLEMENTARY MATERIAL

Williams, T., Ward, K., Egli, V., Mandic, S., Pocock, T., Clark, T. & Smith, M. 2022. More-than-human: A cross-sectional study exploring children's perceptions of health and health-promoting neighbourhoods in Aotearoa New Zealand. International Journal of Environmental Research and Public Health.

Figure S1: Overview of feedback materials for participating schools from the Neighbourhoods and Health study.

<table>
<tr><th>Generation and dissemination of feedback materials to schools</th><th>Example images of dissemination material provided to each participating school</th></tr>
<tr><td>

The full research team and smaller data collection teams met over multiple sessions to generate feedback materials for participating schools. This dissemination activity aimed to provide an overview of the process and outputs that schools could share with participants and the school community, including parents/carers and teachers.

To ensure timely feedback, analysis was brief and involved: 1) MS scanning all material collected, 2) data collection teams from each school generating storylines and key messages, and 3) the whole team collaboratively generating final outputs. Materials were derived directly from participant-generated statements on health and neighbourhoods, and featured children's co-created outputs, ensuring material integration from all groups and individuals.

Each school was provided with a short video and a one page written summary (see example images to the right). Schools were encouraged to share the video at school assemblies, and include the written summary in their school newsletter.

</td><td>

Video:

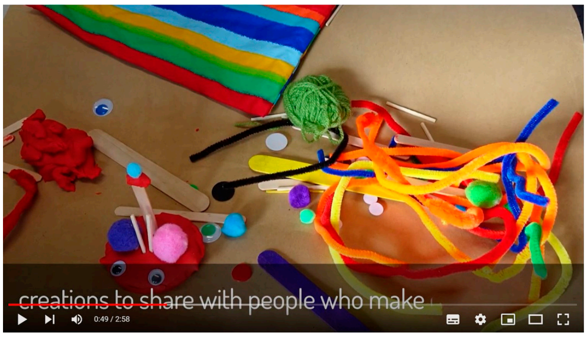

Written summary:

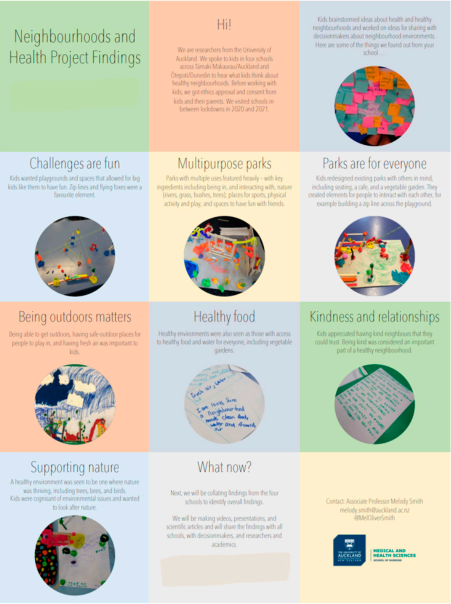

</td></tr>
</table>
